# From Augustine of Hippo’s Memory Systems to Our Modern Taxonomy in Cognitive Psychology and Neuroscience of Memory: A 16-Century Nap of Intuition before Light of Evidence

**DOI:** 10.3390/bs3010021

**Published:** 2012-12-27

**Authors:** Jean-Christophe Cassel, Daniel Cassel, Lilianne Manning

**Affiliations:** 1 UMR 7237, Laboratoire d’Imagerie et de Neurosciences Cognitives, CNRS-University of Strasbourg, IFR 037 des Neurosciences, GDR 2905 du CNRS-12, rue Goethe-F-67000 Strasbourg, France; 2 Lycée André Maurois, 2 rue du Stade, F-67240 Bischwiller, France

**Keywords:** cognitive psychology, declarative memory, emotions, episodic memory, memory, psychology and neuroscience of memory, philosophy, semantic memory, taxonomy of memory systems

## Abstract

Over the last half century, neuropsychologists, cognitive psychologists and cognitive neuroscientists interested in human memory have accumulated evidence showing that there is not one general memory function but a variety of memory systems deserving distinct (but for an organism, complementary) functional entities. The first attempts to organize memory systems within a taxonomic construct are often traced back to the French philosopher Maine de Biran (1766–1824), who, in his book first published in 1803, distinguished *mechanical memory*, *sensitive memory* and *representative memory*, without, however, providing any experimental evidence in support of his view. It turns out, however, that what might be regarded as the first elaborated taxonomic proposal is 14 centuries older and is due to Augustine of Hippo (354–430), also named St Augustine, who, in Book 10 of his *Confessions*, by means of an introspective process that did not aim at organizing memory systems, nevertheless distinguished and commented on *sensible memory, intellectual memory, memory of memories, memory of feelings and passion, and memory of forgetting*. These memories were envisaged as different and complementary instances. In the current study, after a short biographical synopsis of St Augustine, we provide an outline of the philosopher’s contribution, both in terms of questions and answers, and focus on how this contribution almost perfectly fits with several viewpoints of modern psychology and neuroscience of memory about human memory functions, including the notion that episodic autobiographical memory stores events of our personal history in their *what*, *where* and *when* dimensions, and from there enables our mental time travel. It is not at all meant that St Augustine’s elaboration was the basis for the modern taxonomy, but just that the similarity is striking, and that the architecture of our current viewpoints about memory systems might have preexisted as an outstanding intuition in the philosopher’s mind.

## 1. Introduction

What is cognition? In its broadest sense, the term cognition refers to the mental processing of information about both the external world and ourselves. Cognition encompasses the higher functions involved in such processing by, particularly, the central nervous system. In our view, to be considered cognitive, a process must contribute to acquire new knowledge, and make sense of what is perceived, but also to retrieve and utilize memorized information. 

Cognitive functions, therefore, include language, attention, reasoning, planning, initiation and inhibition of responses, problem-solving, anticipating, encoding information, storing and retrieving memories. Originally, the term cognition was restricted to the designation of specifically human mental operations. However, within the cognitive neurosciences framework, taking into account recent research and the emergence of the evolutionary vision of cognition, this term is now also widely used to denote information processing in non human animals. Indeed, it appears that even nematodes such as Caenorhabditis elegans or flies such as Drosophila melanogaster are able to remember an unpleasant experience (e.g., [1,2]). This, however, does not seem sufficient to deduce that they have cognition-driven behaviors. After decades of experimental work, the idea that cognitive processes can be investigated in humans and animals, with, in some instances, comparable methodological principles, is no longer a concern. The same seems to apply to the notion that the structural substrates and the mechanisms therein of cognitive processes may share characteristics among a variety of species. For instance, damage to a structure named the hippocampus alters memory consolidation and spatial navigation behavior in human and non human primates, but also in mice and rats (e.g., [3]).

Our present concept of cognitive functions as well as our ability to measure these functions is the result of the development of methodological issues in Psychology. Indeed, investigations using experimental methods arose only during the 19th century, when the term cognition had not emerged yet and most experimental approaches focused on perception and stimulus-response relationships. Before the 19th century what might be called psychological phenomena were interrogated introspectively, mostly by philosophers. The current study focuses on one of those elaborated theoretical constructions that emerged in the course of introspective reflections. We comment on memory, and even memory systems to use a modern expression, as it was brought to light by St Augustine’s questions, about 16 centuries ago.

Considerable research in normal human memory and in amnesia both from brain damage and neurodegenerative disease has accumulated in the fields of psychology, cognitive psychology and cognitive neurosciences over the past decades. The complementary approaches carried out in these research fields have progressively led to a variety of taxonomies of memory systems, which have proved to have a fruitful heuristic value. Most of these taxonomies are based on a fundamental dichotomy in which conscious encoding/recollection processes are distinguished from non conscious processes, each of which is composed by a series of memory subsystems (e.g., [4,5,6]). There is no consensus, however, about the organization of these systems: some authors distinguish them on the basis of conscious vs. unconscious processes (e.g., [5,6]), when others do not make consciousness a distinction criterion, considering the different systems as highly interactive (e.g., [7,8,9]), and even others advance different positions (e.g., [10]). Debate about which, among the different taxonomies that have been proposed so far most appropriately describes and enables organization of all available experimental and clinical data, as well as our current knowledge, is beyond the main scope of the present study. We aim at focusing on the introspective reflections about memory of the fourth century theologian and philosopher, St Augustine, and try to highlight how most if not all of his contributions do brilliantly fit with aspects of the nowadays well accepted taxonomic constructions proposed by Tulving or Squire and grounded by psychological evidence, both from clinical and experimental viewpoints.

Looking back on the history of memory systems and related taxonomies, it appears that the idea that memory is not a single faculty supported by a single overall functional principle of the mind did not first emerge from empirical approaches, but was already proposed as a hypothetical construction. As stated above, this was done exclusively on the basis of mainly introspective intellectual elaborations by philosophers and psychologists “more than a century ago”, as usually stated in review papers or book chapters. In the Introduction of his relatively recent and comprehensive review, for instance, Squire [5] has referred to a French philosopher named Maine de Biran (1766–1824; [11]). In 1799, and thus long before any empirical approach of memory phenomena had been undertaken (e.g., Hermann Ebbinghaus [12]), Maine de Biran distinguished *mechanical memory*, *sensitive memory* and *representative memory* in close relationship with the memory of habits, the latter term being to be understood in its broadest meaning. His book, entitled *Mémoire sur l'Influence de l’Habitude sur la Faculté de Penser* (Essay on the Influence of Habit on the Faculty of Thinking) was published in 1803 in its French version, and an English translation appeared in 1929.

Although the expressions used by Maine de Biran to name each category of memory appear self-evident, it can be briefly recalled that, in his view, mechanical memory is a memory system involved “*in unconscious learning and repeating sequences of movements or words*”, sensory memory is supporting the most often unconscious acquisition of image—or event-related feelings, and, finally, representative memory, is depicted as the “*nutritive milk of intelligence*” because it is the one to be involved in the conscious recollection of events and images. While this is often one of the earliest traces cited by researchers and theoreticians in cognitive neuroscience in relation to philosophical attempts to build up taxonomic systems, it turns out that, in fact, the first of such introspectively elaborated taxonomies is about 14 centuries older than that proposed by Maine de Biran. We do not assert, however, that it was St Augustine’s deliberate intention to elaborate this taxonomy. He gave a name to what he regarded as different but complementary memory functions, which he metaphorically presented as the palaces of memory in his famous *Confessions* (Book 10). Although St. Augustine did not use the term cognition, his reflections about memory led him to distinguish, beyond a general function that is memory, a variety of memory systems storing and processing different kinds of information.

In the history of philosophy, St. Augustine's ideas were not the first to focus on memory-related questions. About eight centuries earlier, the Greek philosophers Plato (427–348 BC) and Aristotle (384–322 BC) had already written about memory and reminiscence. However, before briefly commenting on their concepts of memory, it is important to acknowledge that the meanings of words like “remembering” or “memory” have changed over the centuries. Danziger [13] reminds us that early on, “remembering” meant listening to a voice and later, it meant looking something up in an inscribed record. Likewise, “memory” was not seen as a universal feature of all human remembering. For Plato, “memory” included an abstract dimension. Importantly, for Plato, the metaphor replaced the myth and memory took its place among natural things and became part of what we now call cognition. Aristotle emphasized, among other aspects, the fact that memory is “*the state of a presentation, related as a likeness to that of which it is a presentation*” and reminiscence is the faculty to “*recover some scientific knowledge which one had before, or some perception, or some other experience, the state of which was declared to be memory*” (Aristotle, in Ross, 1930, [14]). 

Plato's and Aristotle's authority on the question of memory was probably embedded in St Augustine's thoughts. The latter had even attempted to reconcile two viewpoints that do not necessarily merge in a perfect harmony. On the one hand, memory is recognized to be a sensible faculty because it cannot be there if there is no image and it cannot be defined otherwise than being intimately linked to the sensation of time, as had been emphasized by Aristotle (only animals having the sensation of time can have a memory, he stated). On the other hand, St. Augustine did not totally disconnect his thinking from Aristotle's theory of reminiscence, as he recognized memory to be an inheritance of a spiritual power that transports an intrinsic capacity of recognizing a verity (implicit is the idea that this faculty does not necessarily require a prior experience). Although Aristotle interestingly distinguished a “remember what” from a “remember when” and, as St Augustine later recognized that non-human animals have a memory (how would cattle find the way home or birds their nest, if not? said the latter), he did not propose a “taxonomic” organization of memory systems, conversely to St. Augustine, who clearly went a step further along his interrogations. The culminating point of St Augustine's philosophical contribution is probably this brilliant intuition that one of the mostly evolved memory systems one has is actually dealing with personal events—a system we call episodic (or autobiographical) memory nowadays [15]—and that it is precisely this memory system that enables a mental travel in time, whether backwards (when one is visiting one’s past) or forwards (when one is building up one’s projects). Both directions have actually been evoked by St Augustine: this particular kind of memory is necessary to recall one's past, but also to organize one's projects.

Greek mythology and the ensuing philosophical thinking were very likely mastered by St Augustine. Memory was at the core of Ancient Philosophy as the faculty articulating temporal human existence and godly immutability. Mnemosyne—from which *mnèmè* (memory) has been derived, and later on *mens* (mind)—was child of the first parents, heaven (Ouranos) and earth (Gaia), and became by Zeus mother of the nine muses. She was said to be purging mortal life from the mind, freeing from time and unifying with the divine. Mnemosyne enables to transcend time’s evanescence-defining characteristic. Hesiod had her, in his Theogony, singing “*all that was, all that is and all that will be*”, *i.e*., past, present and future, respectively. Time, in all its dimensions, is contained, brought together, and thereby surpassed in and by memory.

At this point, one may guess Augustine’s reflections when seeking God in his *Memoria dei*. St. Augustine was certainly aware of Plato’s theory of reminiscence, in which memory is considered the source of verity. Memory, in Plato’s view, is the capability of retrieving all genuine knowledge, the one that time cannot corrupt and which is buried deeply in the mind. Thus, to know is nothing but remembrance. Indeed, the mind has the property of containing all verities, the latter being nothing but an up surging of the mind’s recollecting its life with the gods, until its incarnation. This reminiscence was St. Augustine’s main source of reference when commenting on knowledge memory in relation to the intrinsic capacity of recognizing the truth. In his last Dialogs, Plato returned to the problem of memory, but in a very different way. Memory was there linked to a sensible faculty and Plato proposed his famous image of memory being like a wax tablet, in which sensations literally imprint themselves into a substrate. The strength of these imprints, and thereby their duration, was thought to be tributary of the malleability of the wax: the more malleable the wax, the less durable the memory!

This image will also be adopted later on by Aristotle. Treading in the footsteps of Plato’s Dialogs, Aristotle’s discarded all mythological background from his reflections, reducing memory to a sensible faculty shared by beings that were capable of feeling time: memory becomes one among several functions of the *Psyche*, and does not seem to have been a major concern for the philosopher. Therefore, one may hypothesize that it is in fact under the influence of Neo-Platonism, and most particularly under that of Plotinus (ca. 205–270 AD) that St Augustine resituated memory a central place within the history of Philosophy. Plotinus considered memory as much more than only a wax tablet in that he saw in it a primordial function that can be differentiated in memory of sensible things, memory of intelligible elements, and memory as a power. In his Enneads (Book IV, Tractates 27 and 28), Plotinus developed the idea of a memory function being intermediate between the world of sensible and the world of intelligible elements. He even exposed a few ideas ahead of their time such as the conception that memories of which we are not aware have a stronger power than those of which we are (an idea very much developed in the whole work of Sigmund Freud later on). Plotinus also wrote “and, in all its memory, the thing it has in mind it is and grows to;” (Ennead IV, 3, 3; [16]).

We might therefore conclude that St Augustine has most probably made use of this already rich critical history of memory, going beyond the oppositions between the different theoretical contributions (Plato *vs*. Aristotle).

## 2. Short Biographical Synopsis of St. Augustine

*Aurelius Augustinus Hipponesis*, also known as Augustine of Hippo or St. Augustine, is best known for his theological and philosophical contributions in the *Confessions* (Ca. 401). He was born in the city of Tagaste (today Souk-Ahras, in Algeria) on November the 13^th^ 354, and died in Hippo (today Annaba, in Algeria) on August the 28th 430, while the Vandals had laid siege to the city, and the Roman Empire was on the way towards its disintegration. While St. Augustine’s father (Patricius) remained a pagan until converting on his deathbed, his mother (St. Monica) was a devoted Christian who provided her son with extensive religious education. 

This education was furthered in the schools of Tagaste until St Augustine was 16 years old. In 370, encouraged by his father, he moved to Carthage (today Carthage, in Tunisia), the city of all pleasures and excesses, where, despite his strong religious education, he succumbed to the temptations. There, in 372, as he later confessed his mother, he had a son, Adeodatus. In 373, he fell into the claws of the Manicheans. Trying as best as they could to reconcile all known religious traditions in their faith, Manicheans preached a dualistic view of the universe, which they considered to be made of two realms, that of light (spirit) and that of darkness (material): Light was the realm of peace, darkness the realm of evil. Under their influence, St. Augustine perfected his education and achieved a full intellectual maturity, which brought him to a progressive rupture with Manichean ideas.

He returned to Tagaste, and later on, he went back to Carthage, in order to teach rhetoric. After 9 years, when almost 30 years old, he definitely turned away from Manichaeism and moved to Italy, first to Rome (383), where he opened a school of rhetoric, and subsequently to Milan (384), where he obtained a professorship. In Milan, he first turned towards pessimistic skepticism, then towards the neo-platonic philosophy. During all these years, and until 386 (the year of the famous episode of the stream of tears under the fig tree), he was constantly torn between his passions (material—darkness in the Manichean view) and his faith (spirit—light). In 387, he was christened by Ambrosius, later lost his mother who had joined him in Milan, and finally returned to his native Tagaste, where he devoted himself to prayer and studying, living an almost monastic life before being ordained priest in Hippo in 391. In 395, he was consecrated bishop of Hippo, where he spent the rest of his life until he succumbed to a fatal illness in his seventy-sixth year.

St. Augustine left a rich work to future generations, in the form of letters (about 220), treatises (about 110), lectures (about 500), essays and books. As the history of ideas has demonstrated, this work has had much more impact in the field of philosophy than any other Christian writer’s work before or after him, with exception perhaps of St. Thomas Aquinas (*ca.* 1225–1274 AC). It is beyond the scope of the present study to provide a comprehensive list of St. Augustin’s works, but among the best known, are *On the City of God* (413–426), *On the Tri*nity (400–416), *Retractions* (426–428), and the most famous and influential of all, his *Confessions* (397–401). 

The *Confessions* consists in a series of Books (1–13). The first of these books to tackle theological and philosophical issues in an exclusive way is Book 10 (books 1–9 have more autobiographical narrative connotations). In book 10, St. Augustine developed his “prescient” view of memory (*the vast domains and palaces of memory*, as he said). He actually distinguished different categories of memories (*the meanderings within the palaces*, as he metaphorically referred to them) based on introspection, and according to various criteria such as the type of content which they process (and which, as concerns his first category termed *“sensible memory”*, are conveyed to the palace by item-dedicated avenues and stored in the palace’s meanderings, at appropriate places, well separated from each other). His conception of memories is not only rich, accurate and detailed, it also is organized within a categorized theoretical framework (*sensible memory, intellectual memory, memory of memories, memory of feelings and passion, and memory of forgetting*); this is corresponding to nothing less than a tentative taxonomy of memory systems, as one would say today. Looking to St. Augustine's writings from a modern position, it turns out that the conceptual distance between his contributions and some of the currently proposed taxonomies (e.g., [5,17]), as they are supported by the huge amount of empirical work that has accumulated over the past 50–60 years in the fields of cognitive neuroscience, psychology and neuroscience of memory, is by far weaker than the temporal distance that separates an outstanding intuition from a much later achieved series of experimental verifications, *i.e*., after about 16 centuries. As documented and discussed herein, although St. Augustine’s reflections did not rely on any scientific evidence and were proposed in a phrasing much different from current neuropsychologists’ and neuroscientists’, this brilliant man totally devoted to faith had eventually described a taxonomy of memory systems which, for most of them, appear to overlap part of some of the memory systems as they are described and debated in our most modern conceptual frameworks (e.g., explicit and implicit memory, episodic memory, semantic memory, perceptual memory…).

## 3. St. Augustine’s “Taxonomic” Framework

Although St. Augustine did not explicitly distinguish taxonomic entities in his reflections about memory, he structured his text, and subsequently reminded this view in a few lines, to make it clear that sensible memory stores only images that enter by the way of our senses, and that knowledge memory stores what we know and which exists by itself once in memory, like in science, or stores notions or notations, which result from all affections of the mind. As such, he focused on what is nowadays considered conscious memory systems by several authors (e.g., [5,6,17]), and it seems that St Augustine did not pay much attention to the question of unconscious memory systems, such as what we call procedural memory.

### 3.1. Sensory Memory (memoria mundi)

**Figure 1:**
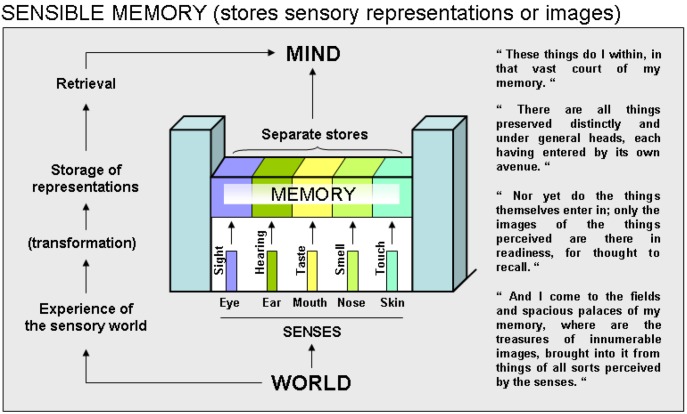
Sensible memory. Based on Book 10 of St. Augustine’s *Confessions*, tentative illustration on how he seems to have considered “sensible memory” in the metaphor of the palace. Column on the right shows relevant quotations of his text. Translations are from E.B. Pusey [18].

A first taxonomic entity proposed by St Augustine (see Figure 1) according to his explanation, is termed “sensory memory”, an entity that Atkinson and Shiffrin recognized in their proposed nomenclature [10]. This memory system, wrote St. Augustine, concerns the storage of sensory representations or images of the world, which the organs of senses contribute to generate. Not do the “realities” actually enter this memory system, only images of perceived realities do so. The eye, the ear, the mouth, the nose and the skin are regarded as the endpoint of avenues, each separately bringing sensation-dedicated perceptions of the world to a first memory palace, which these perceptions enter by the way of sensation-assigned gates (sight, hearing, taste…), and where they will be stored in distinct compartments. From there, they can be recalled to the mind upon request. St. Augustine thought that, these compartments are necessarily separated from each other, as we do not hear light or see sounds when light or sound are actually perceived, nor do we make that kind of confusion when the corresponding memory is retrieved (e.g., we do not remember a sound as light). The sensory memory is specifically dedicated to generate traces of sensory events, which are kept under the form of images that can in no case be confounded with the object as it had been perceived in the external world (this idea, but not in relation with a particular category of memory, was in fact already proposed by Aristotle in his “On memory and Reminiscence” written in 350 BC; [14]).

### 3.2. Memory of Self (memoria sui)

#### 3.2.1. Continuity of the Self

This is the memory in which the subject remembers—and by the same occasion knows—himself. Therein, self experience-related actions, moments, places and impressions are stored. Not any kind of those, but those in which the subject participated as a conscious actor. This is the memory from which one can call his own past, and project into future actions, events and expectations, St. Augustine explained.

#### 3.2.2. Knowledge Memory

**Figure 2:**
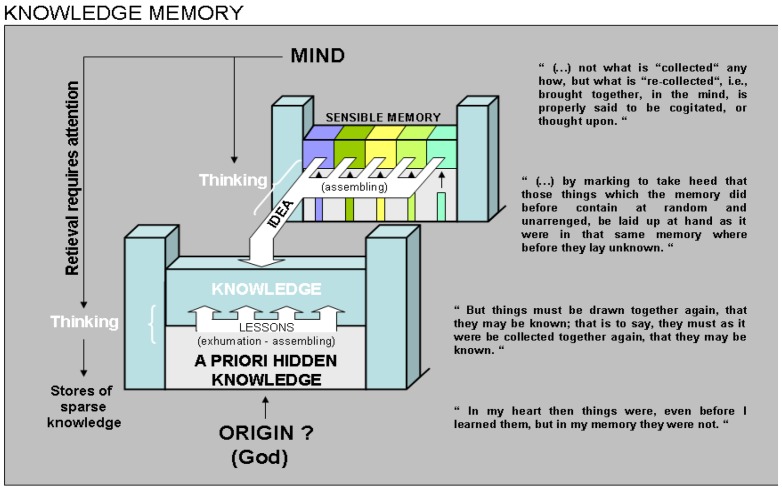
Knowledge memory. Based on Book 10 of St. Augustine’s *Confessions*, tentative illustration on how he seems to have considered “knowledge memory” in the metaphor of the palace. Column on the right shows relevant quotations of his text. Translations are from E.B. Pusey [18].

From the first palace of sensory memory, St. Augustine then distinguishes what could be a second one he called “knowledge memory” or intellectual memory (schematized in Figure 2 following his explanations). In relation to this second memory system, the philosopher made the hypothesis that each subject is born with a store of *a priori* hidden knowledge that is inherited from God (a monotheist view of Aristotle’s idea). There, all things we are going to know already lay as yet unknown, but most probably already in a structured fashion, because, he said, when they come to mind in relation to a particular experience, they seem true to us. To come to mind, which is the *sine qua none* step before these things can be memorized, it is essential that the hidden knowledge be exhumed and assembled. Exhumation and assembling operations are performed during what St. Augustine called the “lessons”, which certainly included teaching but most probably encompassed any kind of information exchange from which knowledge can subsequently be stored and recalled. This memory system, which can also be informed by the way of sounds (as words are sound), images and other sensory modalities, was not supposed to include, however, the image of the ensuing sensations (this is the role of the aforementioned system). Indeed, it is actually storing the signification of things, rules, numbers and measures, their semantics one would say in a contemporary acceptation.

It is because St. Augustine could not figure out a particular door by which these significations entered its body that the philosopher supposed their *a priori* existence in a latent state, from which they had to be “woken up” by experience. This idea was probably inherited from Aristotle’s theory about reminiscence. Furthering his reflections, St. Augustine then proposed that this knowledge is eventually stored as scattered fragments, which the mind has to assemble by the way of thinking, and to keep available by the way of attention. Putting all these things together to bring a given knowledge to mind from the memory where this knowledge is stored in dispersed forms is the specific aim of thinking (*cogitare*). Here, St. Augustine is clearly evocating a kind of associative process, the result of which is nothing but knowledge to be retrieved. This kind of knowledge does not belong to any language, be it Greek or Latin, because it is beyond a given language, he adds.

#### 3.2.3. Memory of Recollection

Augustine carried out another reflection, for which he considered the notion of a memory of recollection, one which is able to remember that one actually already remembered the same thing at earlier occasions. This type of memory is probably an extension of knowledge memory, in that it is dealing with “how” things were acquired when they were. Recalling knowledge memories, stored in a scattered form, was, for the philosopher, an operation requiring an assembling process (see above) before the complete image can be retrieved. Not only is the mind able to reconstruct this image, it does also show the ability of recalling that such an operation has occurred on other occasions and how—or said in a more contemporary way in which context—this former operation had been performed. This is probably the reason why he conceived this memory of recollection, which, however, he did not comment as might have been the case had he considered it an additional entity.

#### 3.2.4. Memory of Feelings and Passions

A fourth memory system concerned the storage of feelings and passions, from which one can retrieve all kinds of experience-related impressions (emotions, one would say nowadays) without having either to feel them when they are retrieved or to be in the same emotional state as the one characteristic of the memory to be able to recall them. For instance, one can retrieve a past sadness while being cheerful or retrieve a past joy while being sad. The mind can be sad when the memory is happy, and vice versa, St. Augustine stated, and there, the philosopher wondered about an only apparent peculiarity of this memory system, as compared to the other systems. Indeed, retrieval of both sensory and knowledge traces brings them to mind in the form of images enabling one to know what it is about. But does the memory of feelings operate in the same way? No reason that it be different. When one speaks about physical pain that is remembered, this pain is not felt. However, if one had no image of that pain, how would one know what it is, and how would one be able to distinguish it from pleasure? Concerning this third system, St. Augustine has not proposed as elaborated layout as he was able to do for the former categories, and especially for his *memoria sui*.

## 4. Memory of Forgotten Things

As for the memory of forgetting, another kind of process he encountered on the way of his reflections, the philosopher became a bit hermetic in some sentences and perhaps even appeared confusing to some extent. He tackled in this section the ability to be conscious that one has actually forgotten something. This particular faculty of the memory might be regarded as one of the aspects of what we call metamemory nowadays (a memory process whose object is awareness about the status of the memory itself, be it still there or lost).

Here St. Augustine is facing an apparent paradox, an “enigma” he asked God to help him to solve: if something is forgotten, how is it possible to be aware that it has gone? Being aware of it would in fact suppose that it is still there, but if still there why cannot it be found in one’s memory? St. Augustine was convinced that memory must have the ability to remember that something has been forgotten. How can it be? From things one tries to remember, and which cannot be found precisely at a given moment despite the fact that one is particularly aware that they should be retrievable, at least that some fragments (of the entire trace) must still be there, he hypothesized. The presence of these fragments do not only help the memory to know that something has been forgotten, it does also help to reconstruct the missing part, and, because they are recognized as such, to discard all false complements of the trace which may try to emerge during this mental reconstruction attempt.

That these intrusive and false complements of the trace are not allowed to complete the available fragment is based on a connection-disabling “mechanism” which operates as long as the reconstructed trace does not match that engraved in one's usual representation. It is not that the appropriate complement of the fragment has gone; were that the case, a reconstruction would never be achieved. It is just that this complement has become less efficiently accessible. St. Augustine proposes an example: if one thinks about or meets a known person and tries to remember this person’s name, which unfortunately does not come to mind, then many names may arise from the memory. The mind, however, will not accept them as the fragments’ (the person) complement, and will do so as long as the appropriate one (the corresponding name) is not found.

## 5. Mirroring St. Augustine’s Memory System Descriptions in Modern Psychology and Neuroscience of Memory

As mentioned in the Introduction and developed afterwards, St Augustine’s Book 10 of *Confessions* is constructed in a way that different types of storage of information are straightforwardly recognizable. In the present section, an attempt is made to highlight parallel ways of thinking between St. Augustine’s and current psychologists’ notions on some memory concepts and systems (see Table 1, below). This attempt is based above all, on the observation that St. Augustine most impressively built up a taxonomic system in terms of *content* of memory and analyzed retrieval. Although he did not separate them formally, he did distinguish what has been called episodic and semantic memory systems 1,575 years later, with no conceptual affiliation whatever between modern psychology and neuroscience of memory and St Augustine's contributions. 

**Table 1:** Synopsis of some possibly parallel concepts in Augustine’s *Confessions*, Book 10, and in modern psychology and neuroscience of memory (PNM).

St. Augustine	Modern PNM [15,21,22,29]
Sensory memory	Semantic memory
—images	—representational
—memories with no bodily sense	—propositionable
Recollections with no context	Noetic consciousness
‘Wondrous cabinets’	Semantic categories ^[28]^
Collected together from dispersion	Binding fragments together ^[26]^
Remembrance	Episodic memory
(when, where, what)	(what, where, when)
Inferring future actions from past experience	A unique system sustaining episodic past and future
Remembering having forgotten	Access memory deficit [46]

Numbers between square brackets indicate corresponding references.

### 5.1. The Context of Book 10

The first characteristic of St. Augustine’s work is that he decidedly set off on long-term, explicit memory reflections. Past memory in the *Confessions* is scrutinized throughout questions, comments and requests addressed to God. The whole work endeavors to know God, therefore, in the Book tackling memory, St. Augustine’s writings were totally guided by his determination to understand how his memory must seek God: *“How do I seek Thee, O Lord? For when I seek Thee, my God, I seek a happy life*” (10, section 20.29; all other quotations are from [18]). Indeed, the context of his meditations is critical to understand why and how he was able to carry out such a high level memory analysis, as *Confessions* is an entirely introspective work.

Book 10 is an earnest enterprise made by an outstanding theologian and philosopher to elucidate how memory of God (memoria Dei) is to be explored: “By remembrance as though I had forgotten it (a happy life), remembering that I had forgotten it? Or desiring to learn it as a thing unknown, either never having known, or so forgotten it, as not even to remember that I had forgotten it? Is not a happy life that all will, and no one altogether wills it not? Where have they known it that they so will it? Where seen it, that they so love it?” (same section). These are some of the central questions that guided the course of St Augustine’s reflections on memory.

### 5.2. The Content of Memory

Psychologists of memory have put forward two types of taxonomies. The first, more intuitive, is based on *time*, from milliseconds to the whole life of an individual (*i.e*., from iconic and echoic immediate memory to long-term memory). The second, more conceptual type of taxonomy applies to long-term memory alone as it is based on *content* of information. William James’s (1842–1910) primary and secondary memory system [19] division is the first taxonomy based on time, and it is still present among us in the form of short-term memory, working memory [20] and long-term memory. Very briefly summarized, the necessary steps imply that attended information gathered from eyes, ears, olfaction, *etc*., enters the sensory immediate memory and undergoes basic processes of identification to be encoded into the short-term store.

This memory, conceptually equivalent to James’s primary memory, needs maintenance rehearsal to allow time for information to be encoded into the long-term memory system, which corresponds to James’s secondary memory. The essence of this notion is that memory is a single capacity equivalent to “storage” and modulated by time. However, this is only part of the story. As Tulving [22,23] pointed out, an important part is how to *retrieve* encoded information. Indeed, contrasting with the straightforward description of memory as storage, whose bases were put forward in 1890, theorizing on different long-term memory contents focusing on retrieval was not proposed until 1972 [15]; see also [23,24]. Only a few years earlier, in the 1960s a scientific meeting explicitly organized to consider the “taxonomy of learning” resulted in all but one participant ignoring the aim of the meeting with the only exception [25] stating that there were no different forms of learning. A few years later, however, Atkinson and Shiffrin [10] presented a model of memory, whose central characteristics are as follows: The authors proposed different categorizations of human memory along different dimensions. The categorization that seems interesting to comment on in the present context is a structural division into three memory systems. The sensory register (SR), short term store and long term store. The SR is defined as the structure that records external inputs in the appropriate sensory dimension. Information is best known for the visual modality, less clear for other modalities. Some amount of information is transferred to the short term system, while the rest is lost. Atkinson and Shiffrin’s SR seems to be the equivalent not only of St. Augustine’s sensory memory but also of the *phantasma* that Aristotle located “in that part of the body that contains the soul” (in Danziger [13]) and which is a copy or likeness (*eikon*) as we mentioned above. 

#### 5.2.1. Constructing Semantic Knowledge

With regard to the storage of sensory information, commented above, we read that St Augustine used this “sensory memory” as a starting point of information processing to be consolidated and retrieved. Thus, in book 10 (section 8.13; [18]), he paved the way—for what we would call today semantic system, semantic knowledge, or memories for facts in Squire’s nomenclature [5]—with the description of acquired basic sensory information (see Figure 1): “There are all things preserved distinctly and under general heads, each having entered by its own avenue: as light, and all colors and forms of bodies by the eyes; by the ears all sorts of sound; (…) All these doth that great harbor of the memory receive…”

He also described in this section, the mental images that are formed on the basis of sensory information that we could liken to the modern concept of representations “Nor yet do the things themselves enter in; only the images of the things perceived”. How those images or representations were formed he confessed not to know, what is beyond doubt is “by which sense each has been brought in and stored up”. Today’s psychologists of memory state that representations concern how the mind symbolizes reality, namely, how and in what ways memory can represent, retain and reconstruct experience [26]. They also state that the stored fragments of sensory experience are encoded in engrams, although, exactly how they are encoded is not completely understood [27].

#### 5.2.2. Retrieving Memories

The notion of binding together stored fragments in order to recollect (reconstruct) a memory has been a cornerstone in some theoretical models. Thus, for example, Damasio’s [28] theoretical framework views encoding of autobiographical incidents as formations of multiple neural configurations, which are located in separate primary sensory and motor cortices. The patterns of neural activity of feature fragments located at that level have combinatorial arrangements, which occurred synchronously during the experience of the event. These patterns of activity are transmitted through downstream neurons to association cortices once they have also been encoded in modality-specific cortical areas. Feed-forward projections towards convergence zones and feedback projections from convergence zones interlock the neural configurations. The model proposes that recall of experiences depends on time-locked neural configuration activations: information stored within the primary cortices is accessed by the activity of the binding codes stored in the amodal convergence zones (association cortices).

Several passages in St. Augustine’s writings announce the process of integrating different stored memory fragments for a recollection to emerge. Thus, in section 10.11.18. [18], he says: “…by conception to receive, and by marking to take heed that those things which the memory did before contain at random and unarranged, be laid up at hand as it were in that same memory where before they lay unknown, scattered and neglected, and so readily occur to the mind familiarized to them. And how many things of this kind does my memory bear which has already been found out (…) and were I for some short space of time to cease to call to mind, they are again so buried (…) for other abode they have none: but they must be drawn together again, that they may be known; that is to say, they must as it were be collected together from their dispersion: whence the word ‘cogitation’ is derived (…) so that, not what is collected anyhow but what is recollected, *i.e*., brought together, in the mind, is properly said to be cogitated, or thought upon.”

#### 5.2.3. Features of Explicit Memories

St Augustine’s memory construction based on integrating scattered information from sensory storage was deepened by the analysis of types of content that he found in the ‘*palaces of memory*’. This resulted in a series of comments that are conceptually close to our notion of semantic knowledge [29], or to that of the memory for facts in Squire’s declarative memory system (e.g., [5]). 

A current and consensual definition of **semantic memory** underscores its characteristic of being memory for general facts of the world, objects and events and other regularities in it. Moreover, Tulving ([29]: see episodic and semantic memory common features) considered that declarative memory (*i.e*., semantic and episodic memory systems) contains stored information that is either representational (isomorphic of what is or could be in the world) or propositionalisable. An Augustinian example of semantic representation memory reads: *“*These things do I within, in that vast court of memory. For there are present with me, heaven, earth, sea…” ([18], 10.8.14). His discussion on representational, sensory-based images, yielded an analogy that could be seen as the first inkling of organized semantic categories [29]: “…images (…) are with an admirable swiftness caught up, and stored as it were in wondrous cabinets, and thence wonderfully by the act of remembering, brought forth”.

Some instances of his reflections concerning propositionalisable semantic contents are as follows: “For what is literature, what the art of disputing, how many kinds of questions there be (…) But the things themselves which are signified by those sounds, I never reached with any sense of my body, nor ever discerned them otherwise than in my mind; yet in my memory have I laid up not their images, but themselves” ([18], 10.10.17). “Here also is all learnt of the liberal sciences and as yet unforgotten” ([18], 10.9.16). “The memory contained also reasons and laws innumerable of numbers and dimensions, none of which had a bodily sense impressed (…) I have heard the sounds of the words whereby when discussed they are denoted: but the sounds are other than the things…” ([18], 10.12.19). 

Tulving’s episodic-semantic distinction was criticized on the bases that his experiments on normal subjects were not methodologically flawless [30]. More particularly, McKoon *et al*. [31] suggested that the episodic-semantic distinction could also be accounted for by the procedural/propositional distinction. However, criticisms seemed to have ended when neuropsychological clinical data were brought to light [32].

#### 5.2.4. To Know But not to Remember

St Augustine added to his reflection on “semantic” memory what could be taken as an expression of noetic consciousness in current memory theory, as mentioned above. Tulving [24,25] introduced and operationalised [25] phenomenological subjective experience accompanying distinct memory systems. Noetic consciousness allows an organism to be aware of and cognitively operate on objects and events in the absence of them. It is therefore expressed without experiencing mental time travel as the context of acquisition is, precisely, irrelevant (and irretrievable) in noetic knowledge. “When and how entered these things into my memory? I know not how. For when I learned them, I gave not credit to another man’s mind, but recognized them in mine; and approving them for true, I commended them to it, laying them up as it were, where I might bring them forth when I willed…” ([18], 10.10.17). Turning back to Tulving’s proposal of declarative memory common features, they include characteristics that are present in this quotation. For example, Tulving states the fact that stored information has truth value (approving them for true), and that behavioral expression is optional (bring them forth when I willed).

At the end of 10.14.22. [18], having carried out a thoroughly scrutiny of the recollection of different passions, St Augustine concluded that recollection of emotions stems, naturally, from experience and that the emotion that accompanies experience can be automatically stored: “And yet could we not speak of them (emotions), did we not find in our memory, not only the sounds of the names according the images impressed by the senses of the body, but notions of the very things themselves which we never received by any avenue of the body, but which the mind itself perceiving by the experience of its own passions, committed to the memory, or the memory of itself retained, without being committed unto it”.

### 5.3. Remembering

At the time when Tulving [15] postulated the distinction between semantic and episodic memory, similar suggestions about different types of memory within both James’s primary and secondary memory had also been proposed. Concerning secondary or long-term memory, only the division of memory in episodic and semantic systems has passed the test of time and it has been steadily investigated for the last four decades. Importantly, there are several sources of convergent evidence that seem to warrant the solidity of such a proposal. Thus, Vargha-Khadem and her co-workers [33,34,35,36] have reported results obtained in groups of developmental amnesic children (showing hippocampal damage sustained perinatally or during childhood), in whom a pronounced dissociation between episodic and semantic memory has been observed. Moreover, in functional neuroimaging research data there is accumulating evidence on a relatively established cerebral network supporting episodic autobiographical memory (e.g., [36]).

The concept of **episodic memory** has naturally evolved since its initial presentation, in 1972 [15]. Tulving had stated that the essence of episodic memory lies in the conjunction of three concepts, self, autonoetic awareness, and subjectively sensed time. His relatively recent definition of episodic memory system (e.g., [22]) is as follows: the human ability to think about personal events (*what*) that happened in another time (*when*), and in another place (where).

In 10.8.14., St. Augustine, having analyzed “semantic” memory, directed his reflections towards memory of the self, akin to the current definition of episodic memory: “There (in the vast court of his memory) also meet I with myself, and recall myself, and when, where, and what I have done, and under what feelings”. The core elements composing the notion of autobiographical memory are present in this outstanding description: the self (I meet with myself), autonoetic consciousness (I recall myself), the temporal and spatial context (when and where), the memory itself (what), and the emotion that accompanies most of episodic retrieval (under what feelings).

In a further section ([18], 10.13.20.), he provided an excellent and detailed analysis of some characteristics of recollections that are most evocative of episodic memory, autonoetic consciousness and subjective time into the past and towards the future: “And I perceive that the present discerning of these things is different from remembering that I oftentimes discerned them, when I often thought about them. I both remember then to have often understood these things; and what I now discern and understand, I lay up in my memory, that hereafter I may remember that I understand it now. So then I remember also to have remembered; as if hereafter I shall call to remembrance that I have now been able to remember these things, by the force of my memory shall I call it to remembrance”.

#### 5.3.1. Combining Past Experiences to Infer the Future

Mentally travel in space is a property of semantic memory in Tulving’s conception, and therefore, it is viewed by him as a precondition for travel in subjective time, which is the hallmark of episodic memory. More precisely, mental time travel allows, through the medium of autonoetic awareness, to remember the self’s previous experiences [29]. Episodic memory is oriented to the past in a unique way as it allows re-experiencing past happenings.

Subjective time does not only cover the past; it also extends into the future. The expansion of the subjective time horizon towards the past in remembering occurred at the same time as an expansion towards the future, stated Tulving [29]. This ability of forward-looking sense of subjective time has been called ‘proscopic chronesthesia’ [37]. Suddendorf and Corballis [38] developed the notion of mental time travel and gave the notion a central place in human mental activity. Their “natural” observation that much of what we talk or write about refers to past and future events makes them suggest that language is strongly associated to mental time travel and, more importantly in relation to our subject, that “mental time travel lies at the heart of human consciousness” ([38], p. 135).

Past-future projection, from a developmental standpoint, has been considered, among others by Povinelli [39]. He indicated that coordination of internal perspectives makes it possible to sustain, besides the current representation of the self, the organization of previous and future representations “under the temporally extended metaconcept of ‘me’”. Taking the coordination of internal perspectives, a step further, Buckner & Carroll [40] within Tulving’s conceptual framework, put forward a strong case for distinct functions that use past experiences for mental exploration of the future (among other departures from the present) and rely on a common set of processes (but see [41]).

From a clinical point of view, future planning ability has been formally tested and reported as being as vulnerable as past episodic retrieval in a few single-case studies [13,21,42,43].

Finally, from a neuroanatomical perspective, it has been demonstrated that future planning is sustained by the same cerebral network that supports episodic memory of past events ([44,45], among others). These results seem to confirm a unique neurocognitive system sustaining personal past recollections and future project evocations, as postulated by Tulving [37].

Several sections in Book 10 illustrate how aware St Augustine was of mental time travel. In 10.8.14., past experience and future evocation are described: “Out of the same store do I myself with the past continually combine fresh and fresh likenesses of things which I have experienced, or from what I have experienced, have believed and hence again infer future actions, events and hopes, and all this again I reflect on, as present. ‘I will do this or that’ say I to myself, in that great receptacle of my mind, stored with the images of things so many and so great, ‘and this or that will follow’. ‘O that this or that might be!’ ‘God avert this or that!’ So speak I to myself: the images of all I speak of are present, out of the same treasury of memory; nor would I speak of any thereof, were the images wanting”

#### 5.3.2. Inaccessible or Lost?

Finally, still another facet of memory that has received St Augustine’s careful analysis is forgetfulness (see Introduction). Interestingly, his final comments on this topic fit with our modern notion of the two suggested types of impairment when retrieval fails: access vs. degradation retrieval deficit of semantic representation. Warrington and Shallice [46] state that access deficit shows in the variability of the patient’s performance due to specific impairment in the procedures required to process a given target. On the other hand, degradation deficit are clearly compatible with a disorder affecting semantic knowledge per se. “For we do not believe it as something new, but, upon recollection, allow what was named to be right. But were it utterly blotted out of the mind, we should not remember it, even when remained. For we have not as yet utterly that, which we remember ourselves to have forgotten. What then we have utterly forgotten, though lost, we cannot even seek after” ([18], 10.19.28).

## 6. Concluding Remarks

About sixteen centuries elapsed from St Augustine’s meditations on memory and the proposition of a multisystem model of memory by Tulving. Modern cognitive neuropsychology of memory has been built on a combined approach of clinical observation and theoretical model proposals. The former was crucial to the beginning of realization that memory is heterogeneous [47]. Cognitive dissociations (as patients’ different performance on questions targeting different functions were named in the 1970s) allowed neuropsychologists to envisage taxonomies based on content. 

How did St Augustine succeed to draw so accurately different memory contents, and did so without any clinical or experimental basis? St Augustine’s interrogations, as they arose while he was seeking God, fed his exploration of each of the “compartments” of memory with concepts and distinctions his spiritual fathers had established: from those compartments that enable human beings to inscribe themselves in the temporal dimension to those on which one may rely for one’s ascension aiming at eternity. His project had two dimensions that his predecessors had opposed, time and eternity. He tried to (re)conciliate them. This objective was not an easy enterprise, and it is probably because it was tremendously difficult that St. Augustine was able to generate different categories of memories both richly detailed and in a fairly short text that we, as authors of the current study, now regard as the first elaborated taxonomy of memory systems.

We turn now to an even more speculative issue: St. Augustine’s view about memory or memory systems in non human animals, as derived from the *Confessions*. St Augustine acknowledged that animals have the faculty of memorizing. “For even beasts and birds have memory; else could they not return to their dens and nests, nor many other things they are used onto” ([18], 10.17). Memory in animals, however, seems to undertake only facts about the body and those images require only sensory memory. Indeed, although not stated explicitly, one has the impression that St Augustine did not consider the other types of memory he distinguished as being shared by humans and other animals. The notion of memory in animals was first discussed by Aristotle. However, in a more metaphoric way, Homer, in the Odyssey and Iliades, made animals more human and humans more animal than will be considered in later views.

As mentioned above, St. Augustine was aware about Aristotle’s and Plato’s contributions. Plato, in his reminiscence theory, stated that memory is proper to humans owing to its implicit ability to allow to “exit” from time and reach the timeless truth of the world of ideas. The platonician reminiscence is built up on Mnemosyne’s role in the mythology: the muse provides humans with the power of escaping the lost of time. It is, therefore, likely that when referring to Plato, St. Augustine did not consider the animals’ memory as an elaborated function. It is noteworthy, however, that in his last writings about his reminiscence theory, Plato recognized a certain kind of memory in animals. Augustine’s introspective research about memory was guided by his desire of happiness by seeking God and, as such, his concern about memory in animals had remained marginal. St. Augustine very likely knew about Aristotle’s zoopsychologie and his suggestion to consider memory as a linear time direction related to sensing time. However, even though Aristotle reintroduced the concept of anamnesis (*i.e*., the effortful retrieval of one’s own story), he made a clear cut dissociation between this anamnesis and memory (*mnèmé*) to which he referred as the power of keeping the past, a power that is common to humans and animals. In “On Memory and Reminiscence”, Aristotle stated that animals with sense perception and awareness of the lapse of time, be they human or not, are capable of memory, while reminiscence is exclusively found in humans. 

For all the aforementioned reasons, most of the different functions of memory carefully browsed by St. Augustin in book 10 of his *Confessions* might have been considered by him as human in nature and, as such, not applicable in extenso to animals. Perhaps memory for senses might have been an exception. Nowadays, driven by experimental evidence, theories have evolved, and expressions like “episodic-like” or “semantic-like” memory systems are used to characterize the abilities thus implied in animals. It is not our intention to state that these memory systems are just equivalent to what the expressions “episodic memory” or “semantic memory” are referring to in humans. However, what scientists call “episodic-like” or “semantic-like” memory in rats, mice and other laboratory animals are often presented as imperfect, incomplete but useful models thereof. Whether they have a genuine scientific value regarding our understanding of human memory systems and the latter’s functional substrates remains a subject of (probably endless) debate.
